# The Split-Half Reliability and Construct Validity of the Virtual Reality-Based Path Integration Task in the Healthy Population

**DOI:** 10.3390/brainsci12121635

**Published:** 2022-11-29

**Authors:** Xiao Fu, Zhenglin Zhang, Yanfei Zhou, Qi Chen, Li-Zhuang Yang, Hai Li

**Affiliations:** 1 Hefei Cancer Hospital, Chinese Academy of Sciences, Hefei 230031, China; 2 Anhui Province Key Laboratory of Medical Physics and Technology, Institute of Health and Medical Technology, Hefei Institutes of Physical Science, Chinese Academy of Sciences, Hefei 230031, China; 3 University of Science and Technology of China, Hefei 230026, China

**Keywords:** virtual reality, entorhinal, hippocampus, reliability

## Abstract

Objective: The virtual reality (VR)-based path integration task shows substantial promise in predicting dementia risk. However, the reliability and validity in healthy populations need further exploration. The present study investigates the relationship between task indicators and brain structures in a healthy population using a VR-based navigation task, particularly the entorhinal cortex (EC) and hippocampus. Methods: Sixty healthy adults were randomly recruited to perform a VR-based path integration task, the digit span task (DST), and an MRI scan. The indicators of the VR-based path integration task were calculated, including the absolute distance error (ADE), degree of angle deviation (DAD), degree of path deviation (DPD), and return time (Time). The reliability of the above indicators was then estimated using the split-half method and Cronbach’s alpha. Correlation and regression analyses were then performed to examine the associations between these indicators and age, general cognitive ability (DST), and brain structural measures. Results: ADE, DAD, and DPD showed reasonable split-half reliability estimates (0.84, 0.81, and 0.72) and nice Cronbach’s alpha estimates (0.90, 0.86, and 0.96). All indicators correlated with age and DST. ADE and DAD were sensitive predictors of hippocampal volume, and return time was a predictor of EC thickness. Conclusion: Our findings demonstrate that the VR-based path integration task exhibits good reliability and validity in the healthy population. The task indicators are age-sensitive, can capture working memory capacity, and are closely related to the integrity of individual EC and hippocampal structures.

## 1. Introduction

Population aging is becoming an increasingly severe global issue. According to the United Nations Population Division, by 2050, the proportion of the elderly in the total population will reach 38% [1]. Mild cognitive impairment (MCI) and dementia will soon become essential diseases affecting public health and economic security [2]. Treatment for cognitive impairment is still in the preclinical stage. The cognitive assessment facilitates early diagnosis and intervention for those at a high risk of dementia [3]. Developing and validating reliable cognitive assessment tools with a solid biological substrate is thus necessary and irreplaceable.

Hippocampal and entorhinal cortex (EC) dysfunction is a potential biological mechanism of cognitive aging [4,5]. Previous studies in animals and humans have shown that the hippocampus and EC take charge of spatial navigation functions, such as pathway integration (PI) [6,7]. PI is the cognitive process that estimates the distance and direction traveled from previously occupied locations, which is essential for spatial navigation. The entorhinal corpus-hippocampus neural circuit helps humans construct spatial cognitive maps and form spatial memory representations of the external world [8]. EC plays a vital role in the pathogenesis of MCI and Alzheimer’s disease (AD). Studies have shown that its structural and functional abnormalities are the precursor symptoms of AD and MCI. However, the hippocampus or medial temporal structure measure shows low sensitivity and specificity than the EC measures. That might explain why popular structural magnetic resonance imaging of the hippocampal cannot help early diagnosis of dementia caused by Alzheimer’s disease in patients with MCI [9]. Therefore, developing behavior measures sensitive to hippocampal, especially the EC’s path integration function, is critical for early diagnosis.

Spatial navigation function, such as PI, is hard to test using the typical paper-and-pencil neuropsychological test. Innovations in the virtual reality (VR) technique offer a promising opportunity. VR enables the creation of an ecological and immersive spatial environment for testing path integration [10]. Most of the previous studies on PI utilize desktop VR techniques. Several studies on healthy populations showed that PI performance decreases with age [11,12]. The PI measures are sensitive to vascular cognitive impairment [13,14]. However, the reliability issue has received insufficient research attention, which is essential for individual difference research. Moreover, although these studies stated that their test measured the hippocampal or EC function, few studies investigated the PI and brain structure measures. 

A recent study by Howett and colleagues has made impressive progress [15]. They developed a new version of the triangle completion task [16] using the head-mounted VR and validated its sensitivity to AD risk. Moreover, they demonstrate the association between the measures of PI and MRI measures of EC volume for the first time in an MCI sample. Although the result is promising, it is still being determined whether this VR path integration task is suitable for aging research in the healthy population. The reliability should be examined before being used as a neuropsychological tool [17]. Furthermore, whether the PI measures of this VR task reflect the EC function and structural integration should be clarified with an independent, healthy aging sample [18].

The present study thus investigates the reliability and validity of the VR-based triangle completion task using a healthy community sample. Split-half reliability is a convenient method to estimate the reliability when test–retest information was not available or a stubborn practice effect existed. We estimate the split-half reliability and Cronbach’s alpha for each behavior measure of PI. High-resolution MRI images are obtained for each subject, and brain structure measures, such as volumes and cortex thickness, are calculated. Our results support the reliability and validity of the VR-based path integration task. 

## 2. Methods

### 2.1. Participants

We recruited nearby community participants by distributing leaflets and posting posters from 2020 to 2022. Sixty-eight participants were recruited. After excluding those who did not complete all tests and had poor MR image quality, 60 people were enrolled (aged 24–84 years). All participants had no history of cancer or dementia, were not taking any medications that could affect cognitive abilities, and had no existing psychiatric or neurological diseases. All participants completed the demographic survey, the VR-based path integration task, the digital span task (DST), and the whole-brain MR scans. The behavior test and MRI scanning interval is no more than one week. Participants were informed of the purpose of this study and the privacy policy before data collection. The study was approved by the Ethics Committee of Hefei Cancer Hospital, Chinese Academy of Sciences. Table 1 provides demographic information.

### 2.2. VR-Based Path Integration Task

All participants wore a wireless VR headset (Oculus Quest, Meta Inc., Menlo Park, CA, USA) and walked in a square of 3.5 m^2^. When they exceeded the safe range, a red boundary warning would appear in their sight, reminding them to stop moving forward. The task was performed in an open virtual environment, and the boundary clues were projected to infinity. The natural and virtual worlds adopt a 1:1 motion ratio to eliminate vestibular mismatches and reduce nausea and other tolerance issues. The task was written in C# language using the Unity 3D engine (version 2019.1.12f1, Unity Technologies, San Francisco, CA, USA) and compiled into an APK file running on the wireless VR headset.

Participants were asked to walk an ‘L’-shaped outward path to three different locations, numbered as 1 (starting point), 2, and 3 (endpoint), flushing on the head. The number of land markers appeared sequentially, and participants walked following the guide of land markers. When reaching the third land marker, a voice will prompt participants to return to the starting location, and all the landmarks disappear. When participants believed they had reached the location, they pressed the button on the handheld controller to end the trial. Before the formal test, there are three practice trials to ensure that participants adapted to the VR environment. We set up three different scenarios in the formal test, each with unique surface details, boundary cues, and lighting. Specifically, one scene has both mountains and lawn surface features, and the other has only mountains or lawn surface features. Each scenario has nine trials. The environmental cues randomly change in each trial. 

**Figure 1:**
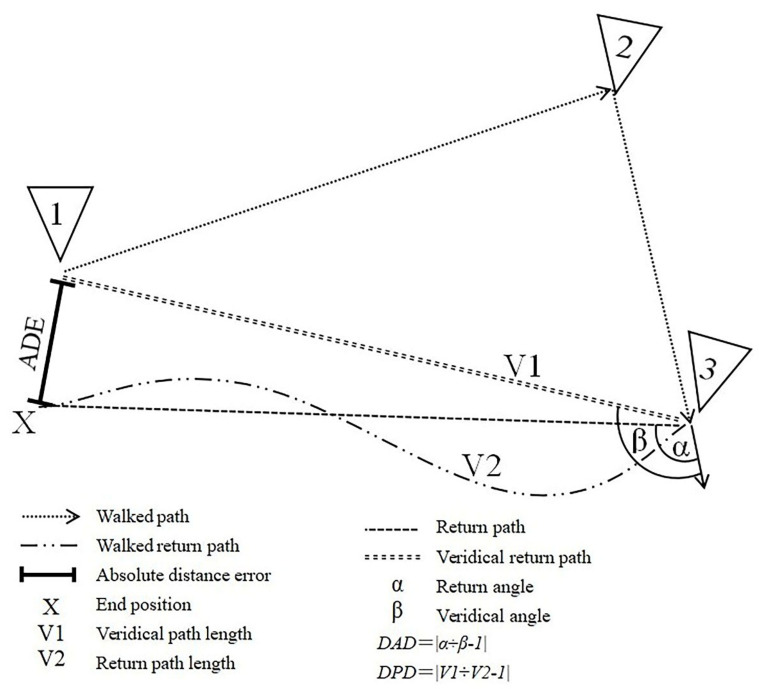
Illustration of the main outcome indicators of VR-based path integration task. VR, virtual reality; ADE, absolute distance error; DAD, degree of angle deviation; DPD, degree of path deviation.

Four PI indicators, absolute distance error (ADE), degree of path deviation (DPD), degree of angle deviation (DAD), and return time (Time), are calculated to evaluate the performance of participants in the spatial navigation task. ADE is defined as the Euclidean distance between the estimated and actual starting positions. DPD reflects the route distance length deviation, and DAD indicates the angle deviation between the estimated and actual positions. The measure of Time is the time taken from the third marker to the estimated position. Figure 1 illustrates the calculation method for those indicators. 

### 2.3. DST

DST is a popular neuropsychological tool to evaluate subjects’ attention and short-term memory. The test includes two parts: forward rehearsal and backward rehearsal. Forward rehearsal contains ten items, and backward rehearsal contains nine items. Each span size includes two number sequences. For each trial, the examiner read each number sequence at the rate of one number per second. The subject then repeat it loudly. Each number sequence counts as one point. The test terminates when subjects fail on two subsequent trials of a particular span-size condition. The forward score is the correct forward sequence number, and the backward score is the number of correct backward sequences. The total score equals the sum of the forward score and backward score.

### 2.4. MR Scanning and Processing

All participants underwent whole-brain MR structural image scans during the experiment using a 3.0 T Philips Achieve system and an 8-channel sense head coil. The 3D-T1-weighted images were obtained in the fast acquisition gradient echo sequence prepared by magnetization. Scanning parameters include voxel size = 1.0 × 1.0 × 1.0 mm3, TR = 7.0 ms, TE = 3.2 ms, matrix size = 256 × 256 pixels, slice thickness = 1.0 mm, scanning time = 4 min and 59 s.

The whole brain segmentation was performed using FreeSurfer 6.0. The cortex parcellations followed the Desikan-Killiany atlas and subcortical segmentation followed the automatic subcortical atlas in FreeSurfer. We used the Desikan-Killiany atlas because it was the default cortex atlas of FreeSurfer, widely used in clinical studies. Two senior radiologists reviewed the images and manually corrected the offsets. Based on previous studies, we selected the EC and hippocampus as regions of interest (ROIs) [19,20]. All brain structure measures were corrected by adjusting intracranial volume.

### 2.5. Statistical Analysis

**Figure 2:**
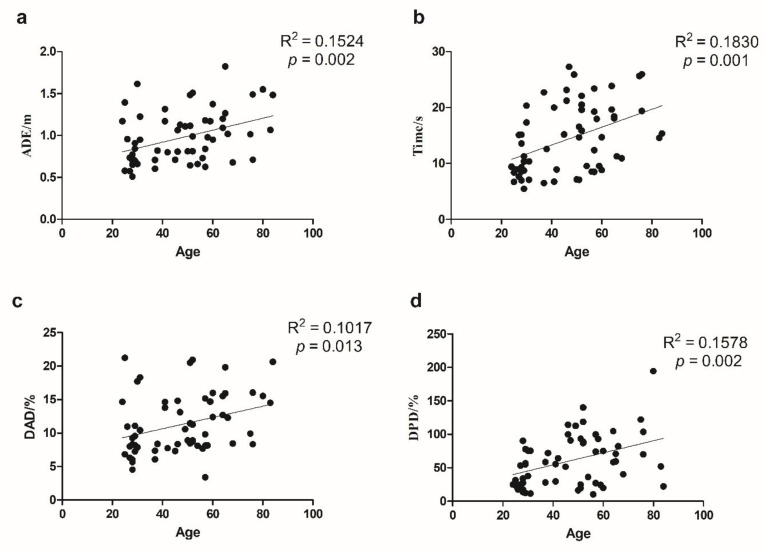
The linear relationship between age and task indicators. (**a**) ADE, absolute distance error and age; (**b**) Time and age; (**c**) DAD, degree of angle deviation and age; (**d**) DPD, degree of path deviation and age.

**Figure 3:**
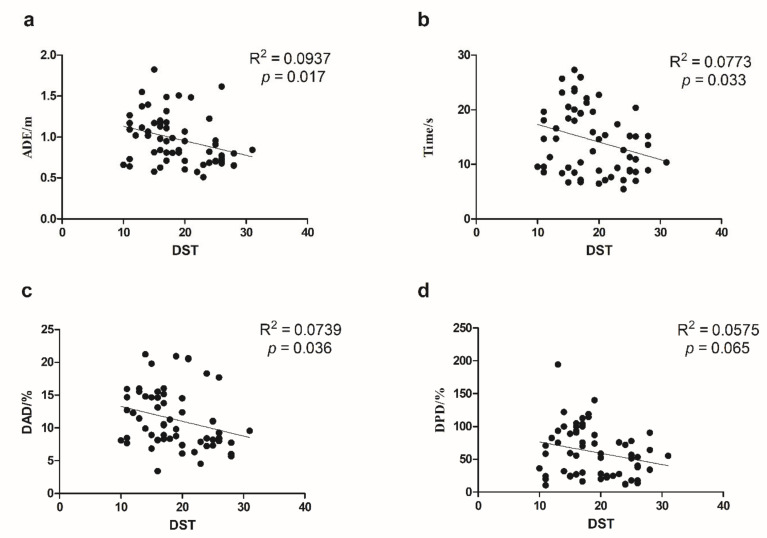
The linear relationship between VR task indicators and DST. VR, virtual reality; DST, digital span task; (**a**) ADE, absolute distance error and DST; (**b**) Time and DST; (**c**) DAD, degree of angle deviation and DST; (**d**) DPD, degree of path deviation and DST.

**Figure 4:**
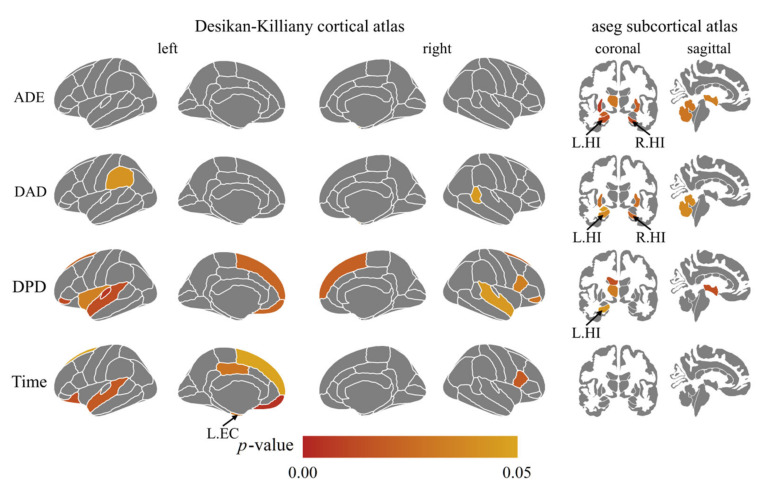
Whole brain correlation analysis on four VR measures. L: left; R: right; HI: hippocampus; EC: entorhinal cortex; The left panel shows the thickness of the cortex, and the right panel shows the volume of the subcortical structure. *p* < 0.05 (Benjamin–Hochberg corrected).

**Figure 5:**
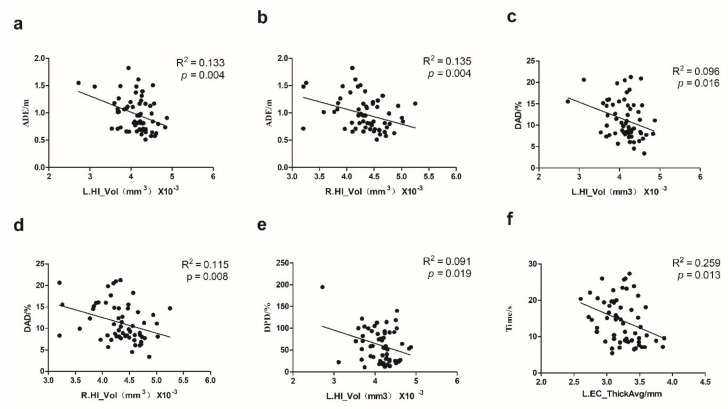
Association between VR task indicators and the structure integrity of HI or EC. L: Left; R: right; HI: hippocampus; EC: entorhinal cortex; Vol: volume; ThickAvg: average thickness. VR, virtual reality. The relationship VR indicators and regions of interest including; (**a**) ADE, absolute distance error and left hippocampus volume; (**b**) ADE and right hippocampus volume; (**c**) DAD, degree of angle deviation and left hippocampus volume; (**d**) DAD and right hippocampus volume; (**e**) DPD, degree of path deviation and left hippocampus volume; (**f**) Time and EC average thickness.

We used SPSS 20.0 (IBM, Armonk, NY, USA) to calculate Cronbach’s Alpha, which is a coefficient representing the consistency of “items” (variables or repetitions) across observations (such as participants). Split-half reliability was calculated using R language and the split-half package [21] using the odd-even split-half method corrected by the Spearman–Brown formula. Regression analysis was conducted using Graphpad Prism (GraphPad Software, San Diego, CA, USA) to investigate the relationship between the indicators of VR-based path integration task, age, and DST(Figure 2 and Figure 3). Pearson’s correlation coefficients among the indicators of VR-based path integration task measures, the cerebral cortex thickness, and volumes of subcortical structures were calculated by R language and corrected by the Benjamin–Hochberg method with *p* < 0.05 was considered significant (Figure 4). GraphPad 8.0 was used to draw the regression analysis diagram (Figure 5). To determine whether the covariate of age drove the VR results and MRI measures, we conducted multiple regression analyses using SPSS 20.0. 

## 3. Results

### 3.1. Reliability of VR-Based Path Integration Task Measures

Table 2 shows the reliability of VR-based path integration task measures. The results indicate that the three measures, ADE, DAD, and DPD, have reasonable split-half reliability and good internal consistency.

### 3.2. Association between Path Integration Measures and Age

Figure 2 illustrates the relationship between age and path integration indicators. ADE, DPD, DAD, and Time increased with age (*adjusted p* < 0.05).

### 3.3. Association between Path Integration Measures and General Cognitive Ability

Figure 3 suggests that the four path integration indicators correlated negatively with DST. ADE, Time, and DAD were significantly related to DST (*p* < 0.05). DPD and DST show a marginally significant association with DST (*p* = 0.065). 

### 3.4. The Association between VR Task Indicators and Brain Structure Measures

We performed a whole-brain correlation analysis of all brain segmentations, the *p*-values of which were corrected for multiple comparisons. Figure 4 illustrates the four VR path integration measures’ associated brain structure measures, which shows that VR path integration measures correlated with thickness or volume measures of the hippocampus and EC. EC and hippocampus were thus selected as regions of interest (ROI) for further analysis, as suggested by previous studies [11,20]. ADE and DAD were negatively related to the left hippocampus volume (*r* = −0.36, *adjusted p* = 0.013; *r* = −0.31, *adjusted p* = 0.038) and the right hippocampus volume (*r* = −0.37, *adjusted p* = 0.012; *r* = −0.34, *adjusted p* = 0.022). DPD was negatively related to the left hippocampus volume (*r* = −0.30, *adjusted p* = 0.043). Time was negatively related to the left EC average thickness (*r* = −0.34, *adjusted p* = 0.03). See Figure 5 for their previous linear regression relationship. To exclude the potential confounding age, we also examined the association between VR task indicators and hippocampus and EC structure integrity with age as a covariate using multiple regression. The trend association between the four VR measures and the hippocampus or EC measure still holds after controlling age (ADE and left hippocampus volume: *p* = 0.074; ADE and right hippocampus volume: *p* = 0.057; DAD and right hippocampus volume: *p* = 0.089; DAD and right hippocampus volume: *p* = 0.043; DPD and left hippocampus volume: *p* = 0.348; Time and left EC thickness: *p* = 0.013).

In addition to the hippocampus and EC ROIs, other brain regions also correlated with VR pathway integration performances. ADE and DAD were negatively related to the right cerebellum-cortex volume(*r* = −0.32, *adjusted p* = 0.02; *r* = −0.31, *adjusted p* = 0.04). DAD was negatively related to the right temporal pole cortex and left supramarginal cortex thickness(*r* = −0.32, *adjusted p* = 0.04; *r* = −0.31, *adjusted p* = 0.04). DPD was negatively related to the left superior frontal cortex thickness, superior temporal cortex thickness, and insula cortex thickness (*r* = −0.34, *adjusted p* = 0.02; *r* = −0.37, *adjusted p* = 0.01; *r* = −0.32, *adjusted p* = 0.03), and right superior frontal cortex thickness, superior temporal cortex thickness, pars opercularis cortex thickness (*r* = −0.36, *adjusted p* = 0.02; r = −0.32, *adjusted p* = 0.04; *r* = −0.34, *adjusted p* = 0.03). Time correlated negatively with the left superior temporal cortex thickness, posterior-cingulate cortex thickness, and superior frontal cortex thickness (r = −0.31, *adjusted p* = 0.02; *r* = −0.34, *adjusted p* = 0.03; *r* = −0.31, *adjusted p* = 0.04) and right pars opercularis cortex thickness (*r* = −0.26, *adjusted p* = 0.02).

## 4. Discussion

The present study investigates the reliability and validity of the VR-based path integration task with a community sample covering young and elderly adults. Our results suggest good reliability of the VR task as evidenced by the split-half reliability estimates and the Cronbach internal consistency estimates. The major VR task indicators decrease with age and correlate with general cognitive ability measured using the DST. Moreover, we demonstrate the association between the VR path integration measure and the structure measures of EC and hippocampus using high-resolution MRI imaging data.

As far as we know, the present study comprises the first reliable evidence of the new VR-based path integration task in the community population. Reliability is essential for individual difference studies. First of all, reliability defines the precision of neuropsychological measurements. The diagnosis must have the reliability information to be trusted. On the other hand, reliability restricts the upper limit of the correlations. Thus, a low-reliability measurement can undermine the power to detect the association with that measurement. Thus, the present study provides a valuable reference for related studies. 

The VR path integration performance is associated with general cognitive ability. Previous studies have demonstrated working memory declines in patients with MCI [22], which continuously worsens in the AD population [23]. The DST, a popular tool for working memory assessment, has been widely used in dementia and MCI research [24]. Previous studies showed that DST is sensitive to AD progression [25]. The present study used a DST to evaluate the construct validity of this task. Our results found that major VR path integration measures correlated with digit span scores, even controlling for the covariates of age. On the one hand, the results indicate that the VR path integration might involve high-level cognitive resources. On the other hand, our findings support the usage of the VR task as a valid measure of cognitive ability. 

The VR-based path integration measures are sensitive to aging. A recent study shows that healthy elderly adults are less efficient in spatial tasks on almost every measure than young adults [26]. For example, when navigation is based on visual or non-visual cues, elderly adults underestimate the distance traveled and the rotation angle in an unfamiliar environment and are less accurate when returning to the starting point than young adults [11,27]. When only vestibular information was available, elderly adults showed poor pathway integration performance compared with young adults [27,28,29,30]. The hippocampus and other medial temporal lobe structures, the critical components of the vestibular system, are particularly vulnerable to aging [31,32,33]. The above studies suggest that the decline of vestibular function [34] plays a significant role in the age-related decline of PI. Therefore, the correlation with age in our study confirmed the reasonable validity of the task.

Our study also verifies the associated biological substrates of path integration measures. The hippocampus can process spatial memory, orientation, and navigation, and the EC’s grid cells and the hippocampus’s location cells jointly construct the spatial cognitive map [8]. The findings of reduced HI volume and EC thickness in patients with aMCI suggest that the virtual path integration task can be used to identify patients with early AD [35]. Studies of healthy adults have shown that the correlation between right posterior hippocampal volume and spatial learning ability is significantly higher in spatially competent individuals than in spatially incompetent individuals [36]. The present study demonstrates that the VR-based path integration task is negatively associated with hippocampal volume and EC thickness in healthy subjects, supporting the usage of the VR path integration task as an indirect measure of hippocampal or EC function.

In addition to the hippocampus and EC, other brain regions are also sensitive to VR path integration measures. First, our results show that ADE and DAD correlated with the right cerebellar cortex thickness, consistent with previous brain imaging studies using mental or virtual navigation tasks [37,38]. The cerebellum helps construct spatial representation maps and aid goal-directed navigation [39]. The cerebral cortex affects how individual neurons interact with each other to constitute a functional brain network[40]. Second, widespread brain regions in the default mode network (DMN) (such as the posterior cingulate cortex and bilateral temporal cortex) and executive network (CEN) (such as Pars opercularis) correlated with path integration performance in the present study. DMN is responsible for episodic memory and self-representation [41,42], which are essential for tracking self-motion and remembering a target location. CEN consists of highly connected hub regions that allow for the adaptive implementation of task demands, linking this network to executive control functions[43,44]. Finally, we also found that DPD correlated with insular cortical thickness. Functional MRI studies of the insular cortex in humans have shown that the insular cortex is involved in cognitive control processes and connects with brain regions involved in sensorimotor processing[45,46], which is vital for self-motion. 

Despite its value to aging researchers, the present study has several limitations. First, we only use the digital span test to examine the construct validity of the VR path integration task. Future studies might use specific spatial ability tests. Standard neuropsychological tests, such as the Mini-Mental State Examination (MMSE) [47], are also necessary. Second, the sample size is insufficient for comprehensive hierarchical analysis. Third, the present study only used brain structure measures. Future studies might use functional MRI to verify the task’s biological validity. 

Another promising future research direction is to examine the relationship between spatial navigation and the sense of agency (SoA). SoA refers to the feeling of control over one’s actions. Previous studies have demonstrated the role of SoA during continuous body movements [48]. In addition, SoA and path integration shared overlapping brain regions such as the premotor cortex and supramarginal gyrus [49,50]. It is thus valuable to test how the SoA interacts with path integration using VR-based spatial tasks. 

## 5. Conclusions

The present study demonstrates that the primary measures of the VR-based path integration task have reasonable split-half reliability and Cronbach’s alpha estimates. These measures are sensitive to age and correlate with general cognitive ability. Moreover, brain structure analysis validates the association between path integration and hippocampal areas. Our results imply that the VR-based spatial navigation test might be a powerful tool in aging research. 

## Figures and Tables

**Table 1:** Demographic characteristics of the study population (*n* = 60).

Characteristics	Mean ± SD
Age	47.6 ± 16.8 (years)
DST	18.9 ± 5.3

SD, standard deviation; DST, digital span task.

**Table 2:** Reliability estimates of VR-based path integration task measures.

	ADE	DAD	DPD
Spearman–Brown	0.84	0.81	0.72
Cronbach’s alpha	0.90	0.86	0.96

VR, virtual reality; ADE, absolute distance error; DAD, degree of angle deviation; DPD, degree of path deviation.

## Data Availability

The data of this study are available from the corresponding author on reasonable request.
